# Spanish Version of the Plymouth Sensory Imagery Questionnaire

**DOI:** 10.3389/fpsyg.2020.00916

**Published:** 2020-05-20

**Authors:** María José Pérez-Fabello, Alfredo Campos

**Affiliations:** ^1^Department of Psychology, University of Vigo, Vigo, Spain; ^2^Department of Psychology, University of Santiago de Compostela, Santiago de Compostela, Spain

**Keywords:** sensory imagery, questionnaire, reliability, validity, mental imagery

## Abstract

**Objective:**

The current interest in mental imagery in fields such as sport and physical training, health, education, underscore the need for designing general measures of imagery vividness that include different sensorial modalities such as the Plymouth Sensory Imagery Questionnaire (Psi-Q; Andrade et al., 2014). The Psi-Q measures imagery vividness in seven sensorial modalities with a factorial structure of seven factors corresponding to the sensorial modalities, and has good internal consistency. The aim of the present study was to translate the Psi-Q into Spanish and to assess its psychometric properties.

**Methods:**

The questionnaire was back-translated, and administered to 394 fine arts undergraduates. Moreover, this test was compared to other questionnaires measuring different types de imagery.

**Results:**

A confirmatory factor analysis found that the Psi-Q had seven factors (vision, sound, smell, taste, touch, bodily sensation, and emotional feeling) with results similar to the original test. Values suggested a better fit for the model of the short version. The internal consistency values were 0.93 for the long and 0.89 for the short test. The Psi-Q subscales correlated significantly (*p* < 0.01) with the total of the Betts’ QMI subscales, and the VVIQ, with the highest significance observed between the Psi-Q Touch and Betts’ QMI Cutaneous (*r* = −0.57), and between the Psi-Q Olfactory and Betts’ QMI Smell (*r* = −0.56). Owing to its novelty, the high correlation and significance (*p* < 0.01) between Psi-Q Vision and the OSIVQ Object (*r* = 0.36) is worth noting.

**Conclusion:**

The Spanish version of the Psi-Q was an adequate measure for evaluating different sensorial modalities of imagery vividness, and exhibited similar psychometric properties to those of the original version. The growing interest in mental imagery in different fields of application justifies the need for adapting the Psi-Q for the Spanish speaking population. This questionnaire is a valuable tool for the understanding of imagery as a psychological process, and as a variable influencing other processes.

## Introduction

Information can be stored and processed by mental imagery. These mental images can be subsequently used in an array of cognitive activities such as thinking, recalling, problem-solving, and daydreaming (Brogaard and Gatzia, 2017). Accessing the content of images is problematic and poses a challenge given that mental imagery constitutes a “private” or “subjective” experience (Richardson, 2005, p. 17). A set of tests were designed to measure an individual’s ability to generate imagery of situations, objects, or people that were not present. Introspection was used to access this content, a method that has been applied to systematically record the verbal reports on a person’s phenomenological experience (see McKelvie, 2019).

In the pioneering work of Galton (1883), the first questionnaire was designed to measure individual differences in the ability to form mental imagery by asking participants to recall, in the most precise manner possible, familiar situations by fundamentally referring to visual imagery, though other sensorial modalities were also used. Subsequently, other quantitative instruments for evaluating mental imagery have been developed, such as the instrument developed by Betts (1909) and the extensively used shorter version (Betts’ Questionnaire Upon Mental Imagery, Betts’ QMI) of Sheehan (1967). This test consists of seven sensorial modalities, and a Spanish version is available (Campos and Pérez-Fabello, 2005). The Cronbach’s alpha of the Betts’ QMI was reported to be good in Campos and Pérez-Fabello (2005), with an alpha of 0.92, and was similar in subsequent studies. Singh and Shejwal (2017) found reliability coefficients ranged from 0.91 to 0.95 in the seven subscales tapping visual, auditory, cutaneous, kinesthetic, gustatory, olfactory, and organic imagery. Miksza et al. (2018) obtained very good internal consistency in the imagery composite scores (0.87), with marginal to good internal consistency for the imagery subscales (visual = 0.79, auditory = 0.72, cutaneous = 0.74, kinesthetic = 0.84, and organic = 0.73). A further widely used questionnaire is the Vividness of Visual Imagery Questionnaire (VVIQ) developed by Marks (1973), which specifically focuses on visual imagery vividness with eyes either open or shut. The Spanish version was designed by Campos et al. (2002). The internal consistency of the VVIQ is good; Campos et al. (2002) obtained a Cronbach’s alpha of 0.88, and McKelvie (1995) a Cronbach’s α of 0.89. The updated version of the VVIQ, which includes 16 items from the original and 16 new items that are presented in a single test and completed with both eyes shut (VVIQ–2; Marks, 1995; McKelvie, 1995), has been evaluated in several Spanish studies (Campos and Pérez-Fabello, 2009; Campos, 2011). Campos (2011) obtained a Cronbach’s α of 0.91, and Campos and Pérez-Fabello (2009) a similar alpha (0.94).

The Plymouth Sensory Imagery Questionnaire (Psi-Q; Andrade et al., 2014) was developed to overcome the psychometric limitations of previous multisensory measures (Betts’ QMI; Betts, 1909; Sheehan, 1967) with factorial structures lacking attested reliability, and in particular, showing a general image vividness factor and weak specific secondary modality factors (for a review of the Betts’ QMI, see McKelvie, 1995; Richardson, 2005; Willander and Baraldi, 2010; Hubbard, 2013, 2018; Lacey and Lawson, 2013). The Psi-Q (Andrade et al., 2014) measures imagery vividness on a range of seven sensorial modalities, with five items for each modality: visual, auditory, taste, touch, bodily sensation, and emotions. The seven factors of sensorial modalities have good internal consistency.

The use of mental imagery in fields such as sports and physical training has led to the proliferation of studies covering a broad range of topics such as Campos et al. (2016), who analyzed the types of mental imagery used in several physical and sports activities. Other authors, Lebon et al. (2019), have investigated motor imagery and its impact on the preparation and execution processes of real movements using transcranial magnetic stimulation (TMS). Zabicki et al., 2019 analysis of spatial patterns of neuronal activity in imaginary actions as measured by functional magnetic resonance imaging (fMRI) revealed significant positive correlations between the subjective impression of motor imagery vividness and objective physiological markers. Rekik et al. (2019) obtained benefits from using dynamic visualizations in the learning of tactical moves in basketball. Ruffino et al. (2019) proposed visualization as a method for preventing the deterioration of motor skills and found that mental training with motor images was beneficial in retaining improvements in performance after physical exercise in the elderly.

Moreover, the use of mental images is increasingly playing a role in physical healthcare. In their study, Grisham et al. (2019) examined the impact of mental images on obsessive–compulsive disorder, taking into account the perspective from which these images were experienced in the field (*first-person*) or observer (*third-person*) perspective. These authors found that higher levels of anxiety were associated with obsessive images experienced in the first person. Saulsman et al. (2019) have underscored the important role of mental imagery in contemporary cognitive behavioral therapy.

An example of the implications of images in education is Birtel et al.’s (2019) study evaluating the effectiveness of an imagery-based strategy designed to reduce prejudice in pre-schoolers. On the subject of music, Loimusalo and Huovinen (2018) examined the silent reading of music to memorize and analyze different types of mental images. Similarly, Pérez-Fabello et al. (2018) analyzed the ability to mentally visualize objects in fine arts, psychology, and engineering undergraduates.

The ongoing research in mental imagery highlights the need to design general measures of imagery vividness that include different sensorial modalities such as the Psi-Q. The review of applied research methods in mental imagery by Roldan (2017) has shown that the Psi-Q was a reliable, valid, and adequate measure for the study of mental imagery and as a first approximation to the analysis of other processes such as perception.

Furthermore, the Psi-Q has been used in several recent studies to measure depression (López-Pérez et al., 2018; Renner et al., 2019), as a method for exploring perceptive diagnostic characteristics using mental visual representations (Roldan, 2017) in mindfulness studies (Kharlas and Frewen, 2016), and in research on dissociative experiences (Denis and Poerio, 2017).

The mounting interest in mental imagery in different fields of application justifies the adaptation of the Psi-Q for the Spanish speaking population by developing a Spanish version of the test. Thus, the aim of the present study was to translate the Psi-Q into Spanish and to assess the structure and stability of the Spanish version of the Plymouth Sensory Imagery Questionnaire (Psi-Q; Andrade et al., 2014), providing a long and short version of the questionnaire as in the original study. Moreover, the long version was compared to the above-mentioned traditional imagery tests for measuring imagery vividness on the VVIQ and the multi-sensory measures on the Betts’ QMI, a more recent test that is extensively used and distinguishes between object, spatial, and verbal imagery (Object-Spatial Imagery and Verbal Questionnaire, OSIVQ; Blazhenkova and Kozhevnikov, 2009). It was postulated that the Spanish version of the Psi-Q would have the same factors as the English version (Andrade et al., 2014). Furthermore, it was hypothesized that there would be strong and significant correlations between the scores of the Spanish version of the Psi-Q and the scores obtained on the VVIQ, Betts’ QMI scales, and the object scale of the OSIVQ.

## Materials and Methods

### Participants

Our sample consisted of 394 undergraduate students from Spain (293 women and 101 men) between 18 and 30 years of age (*M* = 21.01, *SD* = 3.19). The majority (51%) of the students were sophomores, some were freshmen (23.4%), and others were juniors (12.9%) and seniors (12.7%).

### Materials

#### Plymouth Sensory Imagery Questionnaire

The Plymouth Sensory Imagery Questionnaire (Psi-Q; Andrade et al., 2014) has two versions, a long version with a total of 35 items, and a short version with 21 items obtained by deleting the last two items of each sensorial modality of the long version. The questionnaire measures mental imagery vividness related to seven types of sensorial modalities: vision, sound, smell, taste, touch, bodily sensation, and emotions. The questionnaire has five items per sensorial modality, and each item is anchored on a seven-point Likert-type scale ranging between 1 (*no imagery*) and 7 (*imagery as vivid as real-life*). The visual modality refers to appearance (*e.g., “a cat climbing a tree.”*) The auditory modality begins with “imagine the sound of.” (*e.g., “hands clapping in applause.”*) The olfactory modality begins with “imagine the smell of…” (e.g., “a rose.”) The gustatory modality begins with “imagine the taste of…” (*e.g., “a lemon.”*) The cutaneous modality begins with “imagine touching…” (*e.g., “icy water.”*) The corporal sensation modality begins with “imagine the bodily sensation of…” (*e.g., “relaxing in a warm bath.”*) Finally, the emotions modality begins with “imagine feeling” (*e.g., “in love.”*) The total score is calculated by summing all of the items.

The Cronbach’s alpha calculated for the long version of the Psi-Q was 0.92, and it was 0.88 for the short version. The internal consistency for each of the subscales of the Psi-Q was vision = 0.68, sound = 0.77, smell = 0.72, taste = 0.75, touch = 0.75, body = 0.68, and emotions = 0.72. The Cronbach’s alphas of both the long and the short versions of the Psi-Q were marginally lower than those obtained in the original test 0.96 and 0.94 (Andrade et al., 2014), and the Cronbach’s alpha (0.98) found by Denis and Poerio (2017). Kharlas and Frewen (2016) obtained two ranges of internal consistency for two population samples (the α ranged from.78 to.90 for the internet sample and from 0.72 to 0.88 for the undergraduate sample). López-Pérez et al. (2018) analyzed the internal consistency of the scales and obtained the following results: vision = 0.72, sound = 0.86, smell = 0.50, taste = 0.77, touch = 0.86, body = 0.76, and emotions = 0.77.

#### Vividness of Visual Imagery Questionnaire

The Spanish version (Campos et al., 2002) of the Vividness of Visual Imagery Questionnaire (VVIQ) (Marks, 1973) has 16 items that the participants had to complete twice, the first time with eyes open, and the second with eyes shut. An example of an item is: “Visualize a rising sun… The sun is rising above the horizon into a hazy sky.” The items are anchored on a five-point Likert-type scale ranging between 1 (*perfectly clear imagery that is as vivid as the real experience*) and 5 (*no imagery, you only know that you are thinking about the object*). The total score is calculated by summing all of the items. Thus, high scores on the VVIQ indicate low imagery vividness. The Cronbach’s alpha for the VVIQ was 0.93, a good internal consistency and similar to the Cronbach’s alpha of 0.88 obtained by Campos et al. (2002) and the Cronbach’s α of 0.89 found by McKelvie (1995).

#### Betts’ Questionnaire Upon Mental Imagery

The Spanish version (Campos and Pérez-Fabello, 2005) of the Betts’ Questionnaire Upon Mental Imagery (Betts’ QMI) (Sheehan, 1967) was used to examine the vividness of mental imagery in seven sensorial modalities: visual, auditory, cutaneous, kinesthetic, gustatory, olfactory, and organic imagery. The questionnaire has 35 items anchored on a seven-point Likert-type scale ranging between 1 (*Perfectly clear image that is as vivid as the actual experience*) and 7 (*No imagery present at all; you only know that you are thinking about the object*). The total score is calculated by summing all of the items. An example of an item is: “Think of some relative or friend whom you frequently see. The exact contour of their face, head, shoulders, and body.” The higher the scores, the lower the imagery ability. The Cronbach’s alpha for this sample was 0.92, which is on par with the reliability reported by Campos and Pérez-Fabello (2005). Singh and Shejwal (2017) obtained reliability coefficients ranging from 0.91 to 0.95 in the seven subscales tapping visual, auditory, cutaneous, kinesthetic, gustatory, olfactory, and organic imagery. Moreover, Miksza et al. (2018) obtained very good internal consistency for the imagery composite scores, 0.87, with marginal to good internal consistency for the imagery subscales (visual = 0.79, aural = 0.72, cutaneous = 0.74, kinesthetic = 0.84, and organic = 0.73).

#### Object-Spatial Imagery and Verbal Questionnaire

The Spanish version (Campos and Pérez-Fabello, 2011) of the Object-Spatial Imagery and Verbal Questionnaire (OSIVQ; Blazhenkova and Kozhevnikov, 2009) was used to measure three-dimensional cognitive style, and it consists of the object, spatial, and verbal imagery subscales. The items on the object imagery scale and the verbal scale were taken from the Object-Spatial Imagery Questionnaire (OSIVQ; (Blajenkova et al., 2006). Of the 45 items composing the questionnaire, 15 items correspond to a visual cognitive style (*e.g., “My images are very vivid and photographic”*), 15 items correspond to a visual-spatial cognitive style (*e.g., “I can easily imagine and mentally rotate three-dimensional geometric figures”*), and 15 items correspond to a verbal cognitive style (*e.g., “When explaining something, I would rather give verbal explanations than make drawings or sketches”*). Each item is anchored on a five-point Likert-type scale ranging between 1 (*indicates you totally disagree with the statement*) and 5 (*indicates you totally agree with the statement*). The total score is calculated by summing all of the items.

The Cronbach’s alpha for the scales of the OSIVIQ were verbal = 0.77, object = 0.81, spatial = 0.76, and overall = 0.79. The results were similar to those reported in previous studies. Campos and Pérez-Fabello (2011) obtained Cronbach’s alphas of 0.72, 0.77, and 0.81 for the verbal, object, and spatial scales, respectively. Höffler et al. (2017) obtained the following scores: verbal = 0.79, object = 0.93, and spatial = 0.86. More recently, Pérez-Fabello et al. (2018) obtained alphas for the verbal scale = 0.78, the object scale = 0.83, and the spatial scale = 0.82.

### Procedure

The study was conducted in accordance with the ethical rules contained in the Declaration of Helsinki of 2000 and was approved by the ethics committee of our University. Undergraduate students volunteered to participate in the study.

The translation process of the Psi-Q (Andrade et al., 2014) was performed in four steps. First, the first author, who is fluent in English and Spanish, translated the Psi-Q into Spanish. Then, the second author, who was also fluent in English and Spanish, back-translated the Psi-Q back to English without referring to the original version. Third, both authors drafted the final version of the Psi-Q. Finally, both authors and a professional English-to-Spanish translator, who is an expert in psychology, edited the syntax of the items, spelling, and any grammatical errors of the final version of the Psi-Q (see Appendix I).

The Psi-Q, VVIQ, Betts’ QMI, and OSIVQ questionnaires were administered to participants in groups of approximately 20 undergraduates in their usual classrooms. The order of tests was counterbalanced.

### Data Analysis

Statistical analysis was performed using SPSS 25.0 software and IBM SPSS Amos 25. The univariate normality was assessed with the skewness and kurtosis, where indexes close to zero and less than 2 indicate similarity with the normal curve of univariate data (Bollen and Long, 1993; Nuviala et al., 2012). Mardia’s coefficient was used for multivariate normality. According to Bollen (1989), multivariate normality exists when Mardia’s coefficient is less than *p* (p + 2), where *p* is the number of variables observed.

The next step was to calculate the internal consistency of the tests by the Cronbach’s alpha. To test which the hypothesis generated by the original studies was confirmed, confirmatory factor analysis (CFA) was performed using SPSS Amos software, 25 version (IBM), which gives model-fitting indicators (Jöreskog and Sörbom, 1993, 1999). The global fit for models was assessed using six indexes: the χ*2* to degrees of freedom (*df*) ratio—because this index alone is very sensitive to sample size (Jöreskog and Sörbom, 1993)—, the goodness of fit index (GFI), the comparative fit index (CFI), the non-normed fit index (NNFI), the root mean square error of approximation (RMSEA), and the standardized root mean square residual (SRMR). Values of the χ*2* to *df* ratio between 0 and 3 are suggested to indicate a good fit (Bollen and Long, 1993). GFI values above 0.90 are recommended, whilst values equal to 0.95 or higher are recommended for CFI and NNFI (Jöreskog and Sörbom, 1993, 1999; Hu and Bentler, 1999). Values equal to 0.08 or lower are recommended for RMSEA and SRMR (Browne and Cudeck, 1993). Finally, the Pearson product–moment correlation coefficient was used to correlate the Psi-Q with the other imagery tests.

## Results

Table 1 shows basic statistics for each questionnaire for men, women, and total, with similar scores obtained in the different data groups.

**TABLE 1:** Basic statistics of different questionnaires in men, women, and total.

		**Psi-Q**	**VVIQ**	**Betts’QMI**	**OSIVQ**
*M*	Men	181.68	70.46	92.22	135.64
	Women	187.62	72.13	91.35	135.26
	Total	186.38	71.80	91.36	135.56
*SD*	Men	28.11	17.99	24.83	14.69
	Women	27.56	18.98	26.83	17.91
	Total	27.62	19.03	26.18	17.17
*Skewness*	Men	–0.30	0.37	0.65	–0.23
	Women	–0.73	0.48	0.62	–0.07
	Total	–0.62	0.51	0.63	–0.10
*Kurtosis*	Men	–0.53	–0.16	1.12	–0.35
	Women	0.88	0.12	0.98	0.03
	Total	0.45	0.11	1.01	0.04

As for univariate normality, the skewness and Kurtosis indexes of the questionnaires were near zero and below the value of 2. In addition, the univariate normality was calculated by the skewness and kurtosis of each item of the Psi-Q, obtaining values that in most items were near zero and less than the 2, except in the kurtosis index for the values of items 4 (2.71), 5 (2.82), 19 (2.04), and 21 (2.34), which were above the recommended values (Bollen and Long, 1993; Nuviala et al., 2012). Therefore, items 4, 5, 19, and 21 were deleted from both the long and short forms of the Psi-Q. Multivariate normality was confirmed by Mardia’s coefficient (305.09 for the long version, and 114.09 for the short version of the Psi-Q). The data normality obtained justified the use of the maximum likelihood method.

Table 2 shows the standardized coefficients for the long version (31 items) of the proposed model, with values ranging from 0.43 (Item 35) to 0.75 (Item 17). All values were statistically significant (*p* < 0.001). As estimated by the model, the correlations among the seven factors ranged from 0.53 for vision and touch to 0.80 for touch and body. All values were statistically significant (*p* < 0.01).

**TABLE 2:** Standardized regression weights and squared multiple correlations in the confirmatory factor analysis (CFA) of the long form of the PSI-Q.

	**Vision λ x**	**Sound λ x**	**Smell λ x**	**Taste λ x**	**Touch λ x**	**Body λ x**	**Emo λ x**	** δ x**
Item 1	0.72							0.52
Item 2	0.71							0.52
Item 3	0.55							0.30
Item 6		0.54						0.29
Item 7		0.64						0.40
Item 8		0.74						0.55
Item 9		0.63						0.40
Item 10		0.63						0.39
Item 11			0.73					0.54
Item 12			0.67					0.46
Item 13			0.70					0.49
Item 14			0.67					0.45
Item 15			0.55					0.30
Item 16				0.64				0.42
Item 17				0.75				0.56
Item 18				0.61				0.37
Item 20				0.56				0.32
Item 22					0.69			0.48
Item 23					0.69			0.48
Item 24					0.68			0.46
Item 25					0.61			0.37
Item 26						0.57		0.32
Item 27						0.52		0.27
Item 28						0.61		0.37
Item 29						0.56		0.32
Item 30						0.52		0.27
Item 31							0.66	0.43
Item 32							0.69	0.48
Item 33							0.65	0.42
Item 34							0.53	0.28
Item 35							0.43	0.19

The values obtained for the long version suggested an adequate fit for the model with χ*^2^*(733.95), *df* (413), and their ratio 1.78 (*p* < 0.001). Index values were: GFI (0.89), CFI (0.92), and NNFI (0.91). RMSEA and SRMR values were 0.04 and 0.05.

Table 3 shows the standardized coefficients for the short version (20 items) of the proposed model, with values ranging from 0.51 (item 27) to 0.77 (item 11). All values were statistically significant (*p* < 0.001). As estimated by the model, the correlation among the seven factors ranged from 0.42 for sound and taste to 0.74 for touch and body. All values were statistically significant (*p* < 0.01).

**TABLE 3:** Standardized regression weights and squared multiple correlations in confirmatory factor analysis (CFA) of the short form of the Psi-Q.

	**Vision λ x**	**Sound λ x**	**Smell λ x**	**Taste λ x**	**Touch λ x**	**Body λ x**	**Emo λ x**	** δ x**
Item 1	0.73							0.53
Item 2	0.72							0.52
Item 3	0.54							0.29
Item 6		0.58						0.34
Item 7		0.70						0.49
Item 8		0.72						0.52
Item 11			0.77					0.59
Item 12			0.69					0.48
Item 13			0.70					0.49
Item 16				0.70				0.49
Item 17				0.72				0.52
Item 18				0.67				0.45
Item 22					0.72			0.52
Item 23					0.75			0.56
Item 26						0.59		0.35
Item 27						0.51		0.26
Item 28						0.60		0.37
Item 31							0.67	0.45
Item 32							0.75	0.56
Item 33							0.58	0.33

The results of the short version suggested a better fit for the model of the short version with χ*^2^*(216.77), *df*(149), and their ratio 1.46 (*p* < 0.001). Index values were: GFI (0.95), CFI (0.97), and NNFI (0.96). RMSEA and SRMR values were 0.03 and 0.04.

The Psi-Q subscales of the long version correlated significantly (*p* < 0.01) with the total of the Betts’ QMI subscales and the VVIQ, with the highest significance observed between Psi-Q Touch and Betts’ QMI Cutaneous (*r* = −0.55), as well as between Psi-Q Olfactory and Betts’ QMI Smell (*r* = −0.56). The significant correlations (*p* < 0.01) between OSIVQ Object and all Psi-Q scales were also worth noting (see Table 4).

**TABLE 4:** Pearson correlations between the questionnaires and the subscales of the long version of the Psi-Q.

**Questionnaire**	**Psi-Q Vision**	**Psi-Q Sound**	**Psi-Q Smell**	**Psi-Q Taste**	**Psi-Q Touch**	**Psi-Q Body**	**Psi-Q Emo**
Psi-Q Vision							
Psi-Q Sound	0.55**						
Psi-Q Smell	0.58**	0.50**					
Psi-Q Taste	0.36**	0.42**	0.61**				
Psi-Q Touch	0.37**	0.49**	0.56**	0.44**			
Psi-Q Body	0.40**	0.44**	0.53**	0.38**	0.59**		
Psi-Q Emo	0.39**	0.44**	0.50**	0.39**	0.47**	0.48**	
Psi-Q Total	0.64**	0.74**	0.83**	0.71**	0.75**	0.74**	0.72**
VVIQ	−0.33**	−0.36**	−0.41**	−0.30**	−0.38**	−0.39**	−0.38**
Betts’ QMI Visual	−0.37**	−0.35**	−0.40**	−0.27**	−0.33**	−0.36**	−0.38**
Betts’ QMI Auditory	−0.27**	−0.48**	−0.32**	−0.28**	−0.34**	−0.35**	−0.23**
Betts’ QMI Cutaneous	−0.30**	−0.39**	−0.48**	−0.37**	−0.55**	−0.51**	−0.40**
Betts’ QMI Kinesthetic	−0.31**	−0.40**	−0.35**	−0.29**	−0.49**	−0.40**	−0.34**
Betts’ QMI Gustatory	−0.34**	−0.34**	−0.44**	−0.43**	−0.37**	−0.37**	−0.38**
Betts’ QMI Olfactory	−0.27**	−0.36**	−0.56**	−0.41**	−0.39**	−0.43**	−0.39**
Betts’ QMI Organic	−0.26**	−0.35**	−0.37**	−0.22**	−0.34**	−0.37**	−0.39**
Betts’ QMI Total	−0.40**	−0.51**	−0.56**	−0.43**	−0.51**	−0.53**	−0.47**
OSIVQ Object	0.27**	0.19**	0.33**	0.26**	0.24**	0.22**	0.24**
OSIVQ Spatial	0.08	0.07	0.13^∗^	0.07	0.07	0.13^∗^	0.01
OSIVQ Verbal	0.09	0.01	0.06	0.07	0.10	0.15**	0.09
OSIVQ Total	0.24**	0.15**	0.28**	0.21**	0.22**	0.26**	0.19**

Negative correlations were due to the scoring system of each test. *p < 0.05, **p < 0.01.

## Discussion

The need for multidimensional measures of mental imagery is derived from previous studies (for a review refer to Palmiero et al., 2019) that consider mental imagery to be not unitary but rather the product of dynamic representations based on different processing styles. Imagery depends on individual skills and strategies and is enriched by the different sensorial modalities. Our findings revealed that difference in image intensity according to the sensorial modality could be detected by using sensitive and appropriate measures such as the Psi-Q. The results of the present study showed a modified model of the original study of Andrade et al. (2014) in both the long and short versions of the questionnaire. Nevertheless, the data underscored that the model was adequate, particularly the short version. In terms of the correlations with other imagery tests, the coincidence between the highest correlations of the scales referring to the same sensorial organ is worth noting. Another important finding of this study is a high and significant correlation between all Psi-Q scales and the OIVQ Object. Although the object imagery cognitive style, as measured by the OSIVQ object scale, primarily refers to the visual appearance of objects (for a review, Pérez-Fabello et al., 2016), it can also include scenes or situations that involve the other senses (e.g., item 22: *“When reading fiction, I usually form a clear and detailed mental picture of a scene or room that has been described.”*)

The correlations between Psi-Q Body, OSIVQ Spatial, and OSIVQ Verbal were significant but not high. Spatial ability is related to body position and movement in space, and several items on the Body scale of the Psi-Q referred to these situations (see in particular items 27 and 28, *“Walking briskly in the cold”* and *“Jumping into a swimming pool,”* respectively.) This would explain the significant correlations between Psi-Q Body and OSIVQ Spatial. Likewise, OSIVQ Verbal examined preferences in verbal instructions to describe an object or person (e.g., item 36: *“I would rather have a verbal description of an object or person than a picture”*), which would explain the correlations between Psi-Q Body and OSIVQ Verbal. In short, the significant correlations were in agreement with the content of the tests and confirmed the appropriateness of this innovative multisensory questionnaire.

The role of imagery and its significance as a variable in different fields of research is expanding, and this promotes research focused on mental imagery (for a review, see McKelvie, 2019). In spite of the increasing application of new methods in behavioral psychophysics (see, Pearson, 2014), Roldan (2017) has underscored the benefits of combining self-report and physiological measures, noting that the questionnaires provide vast amounts of information, quickly and extensively, and are an initial approach, for example, in studies on cognitive processes such as perception. The benefits of evaluating using both methods were corroborated by the work of Zabicki et al. (2019), who found significant positive correlations between neuronal disparity values and the subjective evaluations of image vividness intensity of the participants, indicating that self-report and physiological measures are complementary.

Hence, this study has shown that the Spanish versions of the Psi-Q are an adequate measure for evaluating different sensorial modalities of imagery vividness and are valuable tools for the understanding of imagery as a psychological process or as a variable influencing other processes (Kharlas and Frewen, 2016; Denis and Poerio, 2017; Roldan, 2017; López-Pérez et al., 2018; Renner et al., 2019). The main limitation of this study was gender differences in the sample; notwithstanding, as the study was not designed to examine gender differences in this variable, this did not compromise the general objective of the study. It is also important to consider many limitations of self-report questionnaires in evaluating data and drawing conclusions (see, for current debates, Archer et al., 2015, 2018a,b; Ioannidis, 2018; Archer and Lavie, 2019).

In short, the Spanish versions of the Psi-Q provide a multimodal measure of imagery intensity that is sensitive and valid for cognitive, neuroscientific, and clinical research, as well as work on imagery. The Psi-Q scores provided evaluations of general and/or specific image vividness for each sensorial modality. As Andrade et al. (2014) pointed out, further research is required to determine the influence of general and specific aspects of each modality of cognitive and neural function on image intensity in each modality. Finally, further studies analyzing the psychometric properties of the Psi-Q in different age groups and cultures are warranted.

## Data Availability Statement

The datasets generated for this study are available on request to the corresponding author.

## Ethics Statement

The studies involving human participants were reviewed and approved by University of Vigo. The patients/participants provided their written informed consent to participate in this study.

## Author Contributions

MP-F and AC contributed conception and design of the study. MP-F organized the database and wrote the first draft of the manuscript. AC performed the statistical analysis and wrote sections of the manuscript. All authors contributed to manuscript revision, read and approved the submitted version.

## Conflict of Interest

The authors declare that the research was conducted in the absence of any commercial or financial relationships that could be construed as a potential conflict of interest.

## Appendix I

*Plymouth Sensory Imagery Questionnaire (Psi-Q)**Cuestionario de Imágenes Sensoriales de Plymouth (Psi-Q)*

*Instrucciones*. El propósito de este test es determinar la viveza de tu imagen en relación a siete modalidades sensoriales: visual, auditiva, olfativa, gustativa, cutánea, sensación corporal y sentimiento emocional. Este cuestionario consta de 35 ítems, 5 ítems para cada modalidad sensorial. Cada modalidad comienza con un título, por ejemplo, para la modalidad visual, “Imagina la apariencia de…”, y a continuación los 5 ítems. Los ítems traerán ciertas imágenes a tu mente y debes puntuar la viveza de cada imagen según una escala de puntuación que tienes a continuación, que va de 1 (sin imagen) a 7 (tan viva como la vida real), siendo 4 la puntuación intermedia. Ten en cuenta que a mayor puntuación mayor viveza. Escribe cada respuesta en el espacio para cada ítem.

Por favor, no pases a la página siguiente hasta que hayas completado los ítems de la página que estás haciendo, y no te fijes en los ítems que ya has hecho para cubrir los posteriores. Recuerda que la escala va de 1 a 7, a mayor puntuación, mayor viveza de imagen. Puedes usar todas las puntuaciones y consultar la escala las veces que necesites.

*Instructions*. The aim of this test is to determine the vividness of your imagery related to seven types of sensorial modalities: vision, sound, smell, taste, touch, bodily sensation, and emotions. The test has 35 items, 5 items per sensorial modality. Each sensorial modality begins with “imagine…,” for example, the visual modality, “Imagine the appearance of…” and then 5 items. The items will bring certain images to your mind, and you are to rate the vividness of each image by reference to the rating scale that is shown below. The scale ranges from 1 (*no image*) to 7 (*perfectly clear and as vivid as the actual experience*); 4 is the intermediate score. Write your answer in the space provided after each item.

Please, do not turn to the next page until you have completed the items on the page you are doing, and do not turn back to check on other items you have done. Try to do each item separately, independent of how you may have done other items. Remember that the scale ranges from 1 to 7, and the highest score is related with the highest vividness. You can use all the scores and check the scale as many times as you need.

Items*Imagina la apariencia de.**(Imagine the appearance of*…*)*

(1) Una hoguera.
  (*A bonfire*)
(2) Una puesta de sol.
  (*A sunset*)
(3) Un gato trepando a un árbol.
  (*A cat climbing a tree*)
(4) Un amigo que conoces bien.
  (*A friend you know well*)
(5) La puerta principal (de entrada) de tu casa.
  (*The front door of your house*)

*Imagina el sonido de.**(Imagine the sound of*…*)*

(6) Una bocina de coche.
  (*The sound of a car horn*)
(7) El palmoteo de las manos aplaudiendo.
  (*Hands clapping in applause*)
(8) Una sirena de una ambulancia.
  (*An ambulance siren*)
(9) Niños jugando.
  (*The sound of children playing*)
(10) El maullido de un gato.
  (*The mewing of a cat*)

*Imagina el olor de.**(Imagine the smell of*…*)*

(11) Hierva recién cortada.
  (*Newly cut grass*)
(12) Madera quemándose.
  (*Burning wood*)
(13) Una rosa.
  (*A rose*)
(14) Pintura fresca.
  (*Fresh paint*)
(15) Una habitación mal ventilada.
  (*A stuffy room*)

*Imagina el sabor de*…*(Imagine the taste of*…*)*

(16) Pimienta negra.
  (*Black pepper*)
(17) Limón.
  (*Lemon*)
(18) Mostaza.
  (*Mustard*)
(19) Pasta de dientes.
  (*Toothpaste*)
(20) Agua de mar.
  (*Sea water*)

*Imagina tocar.**(Imagine touching*…*)*

(21) Piel.
  (*Fur*)
(22) Arena cálida.
  (*Warm sand*)
(23) Una toalla suave.
  (*A soft towel*)
(24) Agua helada.
  (*Icy water*)
(25) La punta de un alfiler.
  (*The point of a pin*)

*Imagina la sensación corporal de*…*(Imagine the bodily sensation of*…*)*

(26) Relajarse en un baño caliente.
  (*Relaxing in a warm bath*)
(27) Caminar enérgicamente en el frío.
  (*Walking briskly in the cold*)
(28) Saltar a una piscina.
  (*Jumping into a swimming pool*)
(29) Tener dolor de garganta.
  (*Having a sore throat*)
(30) Enhebrar una aguja.
  (*Threading a needle*)

*Imagina sentirte*…*(Imagine feeling*…*)*

(31) Emocionado.
  (*Excited*)
(32) Aliviado.
  (*Relieved*)
(33) Asustado.
  (*Scared*)
(34) Furioso.
  (*Furious*)
(35) Enamorado.
  (*In love*)
